# The Pulmonary Artery Catheter in the Perioperative Setting: Should It Still Be Used?

**DOI:** 10.3390/diagnostics12010177

**Published:** 2022-01-12

**Authors:** Thomas Senoner, Corinna Velik-Salchner, Helmuth Tauber

**Affiliations:** Department of Anesthesia and Critical Care Medicine, Medical University Innsbruck, 6020 Innsbruck, Austria; corinna.velik@tirol-kliniken.at (C.V.-S.); helmuth.tauber@tirol-kliniken.at (H.T.)

**Keywords:** pulmonary artery catheter, anesthesiology, critical care, hemodynamic monitoring

## Abstract

The pulmonary artery catheter (PAC) was introduced into clinical practice in the 1970s and was initially used to monitor patients with acute myocardial infarctions. The indications for using the PAC quickly expanded to critically ill patients in the intensive care unit as well as in the perioperative setting in patients undergoing major cardiac and noncardiac surgery. The utilization of the PAC is surrounded by multiple controversies, with literature claiming its benefits in the perioperative setting, and other publications showing no benefit. The right interpretation of the hemodynamic parameters measured by the PAC and its clinical implications are of the utmost essence in order to guide a specific therapy. Even though clinical trials have not shown a reduction in mortality with the use of the PAC, it still remains a valuable tool in a wide variety of clinical settings. In general, the right selection of the patient population (high-risk patients with or without hemodynamic instability undergoing high-risk procedures) as well as the right clinical setting (centers with experience and expertise) are essential in order for the patient to benefit most from PAC use.

## 1. Introduction

Initially, the main purpose of pulmonary artery catheterization, which was first introduced into medicine in 1944 [1], was to assess the severity of mitral valve disease. The pulmonary artery catheter (PAC) was usually inserted into the venous circulation via a large vein in the groin, arm, neck, or chest, and advanced through the chambers of the right side of the heart into the pulmonary artery. It was then further advanced up to the point where the tip temporarily occluded the branch of the pulmonary artery into which it had been inserted. The resulting interruption of forward flow allowed the filling pressures in the left side of the heart to be measured at the end of the occluded pulmonary vessel. The magnitude of the elevated pressure was a measure of the severity of mitral valve disease. At that time, this procedure was carried out mostly in cardiac catheterization laboratories. The introduction of balloon flotation flow-directed catheters by Drs Swan and Ganz in 1970 [2] allowed the insertion of these catheters at the bedside without the need for fluoroscopy. This led to a shift in the role of the PAC as a diagnostic procedure into a monitoring technique with continuous or intermittent measurement of several hemodynamic parameters.

Even though the use of the PAC has declined over the past decades, it remains the preferred invasive hemodynamic method for multiple surgical interventions, such as cardiothoracic procedures including heart transplantation, left ventricular assist device (LVAD) implantation, as well as lung and liver transplants. One of the several advantages of the PAC is the ability to monitor several hemodynamic parameters (continuous cardiac output measurements, right ventricular end-diastolic volume, right heart pressures, pulmonary artery and pulmonary occlusion pressure (also known as “wedge pressure”), pulmonary and peripheral vascular resistance, and mixed venous saturation) as well as therapeutic interventions such as the administration of drugs and right atrial and ventricular pacing [3].

This review will critically examine the role of the PAC in the perioperative setting considering new clinical trials, especially during cardiothoracic surgery.

Take-home messages from the included studies regarding the application of the PAC in various clinical settings are summarized in Table 1.

## 2. The Role of the Pulmonary Artery Catheter in the Perioperative Setting

The PAC allows continuous tracking of several hemodynamic variables, which seems particularly valuable in critically ill high-risk patients with circulatory dysfunction. The measurement of cardiac output can differentiate shock states into hypovolemic etiology (low cardiac output with low filling pressures), cardiogenic etiology (low cardiac output and high filling pressures), and distributive etiology (high cardiac output and low systemic vascular resistance). Furthermore, the measurement of cardiac output and left and right filling pressures (pulmonary artery wedge pressure (PAWP) and central venous pressure (CVP), respectively) allows the distinction between left ventricular (LV) or right ventricular (RV) dysfunction or global dysfunction. For RV dysfunction, the PAC can further differentiate between RV dysfunction predominantly related to increased afterload (high pulmonary artery pressure (PAP)) versus dysfunction related mostly to pump failure (high CVP and low PAP) [10,11].

Despite these advantages, the PAC has seen a decline in its usage, which has been justified with the arguments that it is an expensive, invasive technique which has still not shown to improve patient outcomes. In the ’90s and 2000s, several large, randomized, adequately powered studies have been published regarding the use of the PAC in various settings: general non-cardiac surgery [12], vascular surgery [13], CABG surgery [14], non-surgical patients with congestive heart failure [15], patients with acute lung injury [16], and critically ill patients in the ICU [17]. Overall, these studies have shown no benefit to PAC use, but also no increase in mortality or in hospital or ICU length of stay. It should however be noted that most of these large, randomized studies examined the routine use of PACs and enrolled a sequential cohort of patients, the majority of whom had a relatively moderate risk of death or complications. Furthermore, not all of them employed a specific therapeutic intervention protocol [18]. Indeed, when examining nonrandomized studies which included especially high-risk patients marked either by old age, severe comorbidity, or increased disease acuity, they were able to demonstrate a clinical benefit from PAC use [19,20,21,22].

In 2003, the American Society of Anesthesiology published recommendations pertaining to the perioperative use of PACs [23]. The task force recommended PAC monitoring as appropriate in high-risk surgical patients undergoing high-risk procedures. Furthermore, the specific practice setting as well as the proficiency and experience of clinicians must be taken into account. Finally, the degree of risk for the patient and the risk posed by the procedure itself should influence the decision whether or not a PAC is be used [23].

## 3. The Role of the Pulmonary Artery Catheter in Cardiac Surgery

The evidence-base for PAC use in patients undergoing cardiac surgery is scant, particularly lacking prospective randomized trials, so much of the evidence is derived from retrospective or observational studies. Furthermore, several contradicting studies exist regarding the use of a PAC, with some demonstrating benefits, others showing disadvantages, and still others having neutral results.

A retrospective cohort study [4] including 6844 adults assessed the associations between PAC use in adult cardiac surgery and clinical outcomes. The study consisted of two cohorts, each with 3422 patients undergoing a cardiac procedure with or without the use of a PAC for monitoring purposes. To reduce bias, the patients were matched 1:1 using propensity score matching. Primary outcomes including 30-day in-hospital mortality, length of stay, cardiopulmonary morbidity, and infectious morbidity were assessed. No difference in the 30-day in-hospital mortality could be observed between the two groups; however, PAC use was associated with a decreased length of stay (8.56 days vs. 9.39 days, *p* < 0.001), decreased cardiopulmonary morbidity (OR, 0.87; 95% CI, 0.79–0.96; *p* < 0.001), and increased infectious morbidity (OR, 1.28; 95% CI, 1.10–1.49; *p* < 0.001). Even though no mortality benefit could be observed in this study, a reduced length of stay and decreased cardiopulmonary morbidity, albeit at the cost of an increased infectious morbidity, suggests an overall potential benefit associated with PAC-based monitoring in this population [4].

A large observational study [5] including 11,820 patients undergoing coronary or valvular surgery assessed the impact of PACs on short-term postoperative outcomes compared with standard central venous pressure monitoring. Thirty-nine percent (4605) of the total population had a PAC insertion. Propensity score matching yielded 3519 evenly balanced pairs. After matching, the PAC group had an increased intraoperative red blood cell transfusion (26.3% vs. 23.4%, *p* = 0.004), longer ICU length-of-stay (48 h vs. 39 h, *p* < 0.001), and more postoperative red blood cell transfusion (40.4% vs. 35.5%, *p* < 0.001) compared with the group without PAC insertion. Otherwise, perioperative outcomes were similar between the two groups, including the cardiopulmonary bypass time (*p* = 0.593), ischemic time (*p* = 0.420), stroke (*p* = 0.498), sepsis (*p* = 0.576), and new postoperative renal failure (*p* = 0.563). Operative mortality was 2.4% for both groups and thus not associated with PAC insertion status. The findings in this study suggest that PACs may have limited benefit in cardiac surgery and may even have unintended consequences for postoperative management [5]. 

This study is in contrast to a large retrospective study [6], which included 116,333 patients undergoing PAC placement during cardiac surgery. Overall, PACs were used in 34.4% of all CABG and valve surgeries. Older age (>50 y), American Society of Anesthesiologists classification of three or higher, case duration >6 h, and the presence of a resident physician or certified nurse anesthetist were associated with increased likelihood of PAC placement. A marked (75%) reduction in red blood cell transfusion has been observed in this study in the PAC group compared with the control group (OR 0.23, 95% CI: 0.084–0.64, *p* = 0.0048). No statistically significant differences were noted in the incidence of cardiac arrest or death between groups [6].

Since a clear correlation between right ventricular dysfunction and mortality exists, the use of a PAC to measure the right ventricular function has been used widely in the operating room during cardiac surgery. More recently, 3D-transesophageal echocardiography (TEE) has been increasingly utilized to measure right ventricular volumes. A recent, small, prospective observational study [7] aimed to compare measurements of right ventricular function between 3D TEE and PAC in patients undergoing cardiac surgery. Using both 3D TEE and PAC, right ventricular end-diastolic volume, right ventricular end-systolic volume, stroke volume, and right ventricular ejection fraction were measured. In the assessment of right ventricular function, a high correlation was found between measurements made with a PAC and with 3D TEE [7].

Another small, prospective observational study [8] assessed the correlation between 2D- and 3D-echocardiography-derived cardiac output (CO) with thermodilution-derived CO (TDCO) before and after cardiopulmonary bypass (CPB). Seventy-eight patients undergoing CPB were included in the study, and advanced hemodynamic variables, including CO by 2D and 3D TEE and a thermodilution technique using PAC, were collected. The 2D CO, 3D CO-diameter, and 3D CO-area values pre-CPB highly correlated with one another, while echocardiography-derived measurements were only modestly correlated with TDCO measurements. A similar pattern could be observed for post-CPB values. Even though significant correlation among 2D CO, 3D CO-diameter, 3D CO-area, and TDCO in both the pre-CPB and post-CPB periods existed, the limits of agreement were wide, suggesting that 2D- and 3D-derived measurements are not interchangeable with those obtained by thermodilution. Therefore, although echocardiography-derived CO may be useful as a monitor in these patients, the present study did not support replacing PAC measurements with echocardiography [8].

Raymond et.al. [24] described in 2019 perioperative right ventricular pressure monitoring in cardiac surgery, especially in patients scheduled for LVAD-implantation and heart transplantation, a patient population which has an up to 30% risk of developing RV failure [25]. Right ventricular pressure is monitored by a special thermodilution paceport pulmonary artery catheter with a pressure transducer connected to a port located 19 cm from the distal tip of the PAC. RV pressure waveform analysis by a PAC can facilitate the diagnosis of RV diastolic dysfunction, with a particular focus on the diastolic component, as well as RV outflow tract obstruction with a focus on the systolic gradient between right ventricular and pulmonary artery pressure. RV pressure monitoring by PAC is a suitable additional monitoring device to echocardiography. The main advantage is its simple and continuous evaluation of RV dysfunction. Continuous RV pressure waveform monitoring can be used as a guide for therapeutic effects on RV function like fluid administration or removal, NO inhalation, administration of milrinone or levosimendan, or cardiac pacing.

## 4. The Role of the Pulmonary Artery Catheter in Liver Transplantation

Advanced cardiovascular monitoring, such as with a PAC, has routinely been used in clinical practice to manage patients undergoing liver surgery. About 8% of patients with chronic liver disease have underlying portopulmonary hypertension, and in patients with severe pulmonary hypertension (mean pulmonary artery pressure >50 mmHg) mortality in the perioperative period during liver transplantation approaches 100%. The PAC may be useful in differentiating the causes of pulmonary hypertension, including high cardiac output and low vascular resistance states and increased blood volume with high pulmonary artery occlusion pressure, which result in a better prognosis than patients with elevated pulmonary vascular resistance [26].

A recent retrospective study [9] included data from 316 liver transplant operations and compared PAC use alone, TEE alone, or the combination of both methods, on the length of hospitalization, 30-day mortality, and various other outcomes. Patients in the TEE + PAC group had the shortest median length of hospitalization (8.6 days; *p* = 0.03) and the lowest 30-day mortality rate (1.3%; *p* = 0.047). However, the TEE + PAC group also had the highest rate of a new postoperative need for dialysis (TEE + PAC, n = 8 [10.3%]; TEE, n = 2 [5.1%]; PAC, n = 1 [0.5%]; *p* < 0.001). When TEE and PAC are being compared with each other, PAC use was associated with a shorter length of hospitalization (9.1 vs. 10.3 days) as well as a lower 30-day mortality rate (3.5% vs. 12.8%). This study showed that the combination of PAC and TEE leads to better outcomes in terms of the length of hospitalization and 30-day mortality, albeit at the cost of an increased need for dialysis postoperatively [9]. 

## 5. The Role of the Pulmonary Artery Catheter in Lung Transplantation

Even though there is a paucity of data on the use of the PAC in lung surgery, its routine use is still common in the western world. Severe pulmonary hypertension is one of the most common indications for PAC use, given its high reliability in measuring beat-to-beat pulmonary artery pressure in the operating room [27]. Besides being able to assess the level of pulmonary hypertension present, the PAC can also determine the RV function, which has been shown to be a strong predictor of survival [28].

## 6. The Role of the Pulmonary Artery Catheter in Patients with Circulatory Shock

The ability of the PAC to measure several important hemodynamic variables as well as tissue perfusion variables (e.g., mixed venous oxygen saturation (SvO_2_), oxygen utilization, oxygen delivery, oxygen extraction, and PvCO_2_) makes it especially useful in the management of patients with shock. As such, current guidelines recommend the use of the PAC over other less invasive devices in patients with severe or refractory shock to monitor cardiac function and CO [29]. Furthermore, a contemporary, large study assessing PAC use and outcomes in cardiogenic shock showed a reduced in-hospital mortality in patients in whom PAC-derived hemodynamic data were used prior to mechanical circulatory support initiation [30].

## 7. Comparison of Different Cardiac Output Monitoring Devices with PAC

Since the introduction of the pulmonary artery catheter into clinical practice, routine measurement of CO has become available at the bedside. Since then, several less-invasive CO-monitoring devices have been developed (Table 2). Non-calibrated pulse contour systems estimate CO based on pulse contour analysis of the arterial waveform, and thus require only a conventional arterial line to obtain an input signal [31]. One such device is the LiDCO hemodynamic monitor, which measures CO continuously, via blood pressure, over an arterial line. It can be set up in a short amount of time and can be used in the whole spectrum of the perioperative setting. Disadvantages of this device are similar to other devices using an arterial line, such as arterial waveform artifacts or arrhythmias compromising data accuracy. Unique to the LiDCO monitor is the usage of lithium chloride, which makes it not suitable to use in patients undergoing treatment with lithium salts. Another important limitation of the LiDCO monitor is the fact that the calibration is affected by neuromuscular blockers [32]. The PiCCO (Pulse index Continuous Cardiac Output) is another device which integrates a wide array of static and dynamic hemodynamic data through a combination of trans-cardiopulmonary thermodilution and pulse contour analysis. As compared to the LiDCO monitor, the PiCCO necessitates intra-arterial (usually using a large artery such as the femoral, brachial, or axillary artery) and central venous catheterization, which limits its broad clinical use [33]. TEE is another non-invasive device which is being increasingly used intraoperatively to determine CO. CO measurement by TEE can be achieved by non-Doppler or Doppler-based methods. In clinical practice a Doppler-based method is commonly used [34]. TEE has been shown to be a reliable tool to assess significant CO changes [35]. Apart from that, it has several other clinically important advantages, as it can be used perioperatively to assess cardiac anatomy and function, preload, and myocardial ischemia, among other features. As compared to transthoracic and epicardial echocardiography techniques, TEE probe manipulations are restricted within the confines of the esophagus and gastrium. As a consequence, it necessitates complex and technically difficult probe manipulations, and thus requires significant training and expertise [36].

## 8. Complications

Insertion of a PAC can be accompanied by several complications, which can be mainly divided into seven categories as shown in Box 1. Many of these complications are relatively uncommon and preventable [41].

Box 1 **Complications of pulmonary artery catheter insertion.**

- Complicated vascular access (pneumothorax, hematoma).
- Arrhythmias (e.g., heart block, ventricular tachycardia/fibrillation).
- Catheter knotting.
- Pulmonary thrombosis and infarction.
- Endothelial or valvular damage.
- Colonization and bacteremia.
- Pulmonary artery rupture.

Complications arising from venous access are the same as those occurring during the insertion of a central venous catheter. In a multicenter study comparing hemodynamic management guided by a PAC versus a central venous catheter, complications were uncommon and were similar in both groups. Positive blood cultures following catheter insertion were found equally often in both groups [16]. Cardiac arrhythmias are a common occurrence, albeit usually benign, except in moribund patients. Prophylactic use of lidocaine to prevent arrhythmias has been suggested in predisposed patients, but its routine use cannot be recommended [42]. Patients with left bundle branch block may develop complete atrioventricular block following PAC insertion, but this is exceptionally rare. If the catheter is advanced carefully and the presence of the catheter in the pulmonary artery is confirmed before further advancement into the right ventricle, knot formation will be rare. Special care should be taken not to advance the catheter by more than 30 to 35 cm into the right ventricle. With the development of heparin-coated catheters, thrombotic complications have become rare. The occurrence of an infiltrate beyond the tip of the PAC on the chest film should raise the suspicion of an evolving thrombotic event, which should lead the physician to consider withdrawal of the catheter. Endothelial damage has primarily been described in autopsy reports, and its clinical relevance is doubtful. Endocarditis following PAC insertion is very rare. Valvular damage mainly occurs due to improper handling of the catheter (especially when withdrawing the catheter while the balloon is still inflated). As applies to any patient with an indwelling catheter, catheter-related infections also occur with PACs, but they do not seem to be any more common than with central venous catheters [41].

Pulmonary artery rupture, although an exceptionally rare complication, is probably the most feared complication due to its high mortality rate (70%) [43]. Overinflating the balloon in the presence of resistance to inflation, especially in the presence of preexisting pulmonary artery hypertension, represents the most common cause. The development of hemoptysis constitutes the cardinal sign of rupture. In such an event, the catheter should not be completely removed, but rather withdrawn slightly followed by the inflation of the balloon. In the case of sustained hemorrhage, a thoracotomy may be necessary to repair the pulmonary artery [41].

## 9. Conclusions

When analyzing the current data published on the use of PACs, a reasonable conclusion is that PAC use is reserved to centers with significant experience and expertise. The PAC generally is used to monitor and guide therapy in high-risk surgical patients undergoing high-risk procedures, those at high risk for hemodynamic instability, and those who are judged more critically ill by a variety of clinical means, especially if elderly and suffering from other systemic diseases.

There exist a variety of less invasive alternatives to the PAC for CO measurement with their respective advantages and disadvantages (Table 2). Probably the clinically most important alternative to the PAC, especially for intraoperative hemodynamic monitoring, is echocardiography as the most common form of non-invasive cardiac imaging. Apart from the fact that the accurancy of TEE is highly dependent on the quality of the echocardiographic images and the operator’s skills and experience, a great disadvantage is still the fact that it does not allow safe, continuous monitoring over hours or days.

In conclusion, in the perioperative setting PAC remains the gold standard for CO measurement. Obviously, the PAC itself has no potential for benefit unless it guides therapies that improve patient outcomes. Future research should focus on defining subgroups of patients who might benefit from use of a PAC, as well as defining effective therapeutic interventions based on the hemodynamic information gained from the PAC. 

## Figures and Tables

**Table 1:** Take-home messages from the included studies regarding the clinical application of the pulmonary artery catheter.

Study Design	Clinical Setting	Sample	Measures	Outcome
Retrospective cohort study [4]	Cardiac surgery	6844 patients, divided into 2 cohorts with or without PAC	Primary outcome: 30-day in-hospital mortality, hospital LOS, cardiopulmonary morbidity, infectious morbidity Exploratory outcomes: AKI, gastrointestinal complication, liver complication, neurologic complication, SOFA CV unplanned readmissions, and all-cause readmissions	PAC use was associated with ↓ hospital LOS, ↓ cardiopulmonary morbidity, ↑ infectious morbidity
Observational study [5]	Cardiac surgery	11,820 patients undergoing coronary or valvular surgery; PAC versus standard CVP monitoring	Impact of PAC on short-term postoperative outcomes (operative mortality, ICU length of stay, stroke, sepsis, renal failure, RBC transfusion)	PAC group had ↑ intraoperative RBC transfusion, longer ICU length of stay, and ↑ postoperative RBC transfusion
Retrospective study [6]	Cardiac surgery	116,333 patients undergoing PAC placement during cardiac surgery	Intraoperative outcomes: death, cardiac arrest, RBC transfusions	PAC use was associated with a ↓RBC transfusion; death and cardiac arrest cases were similar between the two groups, although a trend towards ↓ mortality could be observed in the PAC group
Prospective observational study [7]	Cardiac surgery	31 patients undergoing elective cardiac surgery with PAC monitoring	Compared measurements of RV function between 3D TEE and PAC	A high correlation was found between measurements made with a PAC and with 3D TEE
Prospective observational study [8]	Cardiac surgery	78 patients undergoing elective cardiac surgery	Correlation between 2D- and 3D-echocardiography-derived CO with thermodilution-derived CO before and after CPB	2D- and 3D-derived measurements are not interchangeable with PAC measurements; this study did not support replacing PAC measurements with echocardiography
Retrospective study [9]	Liver surgery	316 patients undergoing liver transplantation who were monitored intraoperatively with TEE alone, PAC alone, or both methods	Total hospital LOS, ICU LOS, need for postoperative mechanical ventilation, new postoperative need for dialysis, postoperative myocardial ischemia, cerebrovascular complication, return to the operating room within 7 days of transplant, and death within 30 days of transplant	TEE + PAC associated with ↓ length of hospitalization and 30-day mortality rate but ↑ new postoperative need for dialysis; PAC vs. TEE associated with ↓ length of hospitalization and 30-day mortality rate

Abbreviations: AKI: acute kidney injury; CO: cardiac output; CPB: cardiopulmonary bypass; CVP: central venous pressure; ICU: intensive care unit; LOS: length of stay; PAC: pulmonary artery catheter; RBC: red blood cell; RV: right ventricle; TEE: transesophageal echocardiography; ↓: decrease; ↑: increase.

**Table 2:** Comparison of Different Cardiac Output Monitoring Devices Regarding Their Advantages and Disadvantages.

Device	Type	Advantages	Disadvantages
PAC [37]	Invasive	The ability to measure several hemodynamic parameters beyond CO	Complications associated with insertion of the catheter Invasiveness
Continuous CO by PAC [37]	Invasive	Continuous CO measurement The ability to measure several hemodynamic parameters beyond CO	Complications associated with insertion of the catheter Invasiveness
PiCCO [33]	Minimally invasive	Continuous CO measurement	Arterial waveform artifact may significantly affect data accuracy Requires transpulmonary thermodilution calibration Requires intra-arterial and central venous access Inability to measure pulmonary artery pressures
LiDCO [32]	Minimally invasive	Continuous CO measurement Useful in goal-directed therapy	Arterial waveform artifact may significantly affect data accuracy Irregular pulse rate may affect data accuracy Calibration affected by neuromuscular blockers Contraindicated in lithium therapy Requires transpulmonary lithium dilution calibration Inability to measure pulmonary artery pressures or extravascular lung water
FloTrac [38]	Minimally invasive	Continuous CO measurement	Arterial waveform artifact may significantly affect data accuracy CO measurements are not accurate enough for use in patients with septic shock, advanced liver disease and other medical conditions associated with decreased vascular tone.
PRAM [39]	Minimally invasive	Continuous CO measurement	Intra-arterial catheter required for reliable trace Technical complications (e.g., over- or under-damping of arterial waveforms) Patient-related complications (e.g., arrhythmias, aortic valve pathology, mechanical assist device)
TEE [40]	Minimally invasive	Useful in the evaluation of cardiac anatomy and function, preload, and myocardial ischemia	Significant training and experience required Accurate readings are operator dependent Not continuous Time consuming
ED [37]	Minimally invasive	Useful in goal-directed therapy	Measures flow only in descending thoracic aorta Assumptions about aortic size may not be accurate Accurate readings are operator dependent

Abbreviations: CO: cardiac output; ED: esophageal doppler; LiDCO: lithium dilution; PAC: pulmonary artery catheter; PiCCO: Pulse index Continuous Cardiac Output; PRAM: pressure recording analytic method; TEE: transesophageal echocardiography.

## Data Availability

The data presented in this study are openly available in PubMed.
